# Proposed salvage treatment strategy for biochemical failure after radical prostatectomy in patients with prostate cancer: a retrospective study

**DOI:** 10.1186/1748-717X-9-208

**Published:** 2014-10-20

**Authors:** Makito Miyake, Nobumichi Tanaka, Isao Asakawa, Yosuke Morizawa, Satoshi Anai, Kazumasa Torimoto, Katsuya Aoki, Tatsuo Yoneda, Masatoshi Hasegawa, Noboru Konishi, Kiyohide Fujimoto

**Affiliations:** Department of Urology, Nara Medical University, 840 Shijo-cho, Nara, 634-8522 Japan; Department of Radiation Oncology, Nara Medical University, 840 Shijo-cho, Nara, 634-8522 Japan; Department of Pathology, Nara Medical University, 840 Shijo-cho, Nara, 634-8522 Japan

**Keywords:** Prostate cancer, Biochemical failure, Salvage radiotherapy, Prostate-specific antigen, PSA doubling time, PSA velocity

## Abstract

**Background:**

Treatment options for patients with recurrent disease after radical prostatectomy include salvage radiotherapy of the prostatic bed and/or androgen deprivation therapy. To establish an effective treatment strategy for recurrent disease after radical prostatectomy, we retrospectively analyzed the outcome of salvage radiation monotherapy in such cases.

**Methods:**

Data from 61 men who had undergone salvage radiation monotherapy for biochemical recurrent disease after radical prostatectomy were retrospectively reviewed. In all patients, salvage radiotherapy consisted of iraradiation to the prostatic bed (70 Gy) using three-dimensional conformal radiotherapy techniques. Treatment outcome was analyzed to identify predictive factors of salvage radiotherapy.

**Results:**

The biochemical recurrence-free survival after salvage radiation monotherapy at 2 and 5 years was 55% and 38%, respectively. Cox proportional regression models revealed that the independent predictive factors for biochemical recurrence were Gleason Score ≥ 8, negative surgical margin, and PSA velocity ≥ 0.38 ng/mL/year. Negative surgical margin and PSA velocity ≥ 0.8 ng/mL/year were significantly associated with poor response in the serum PSA levels after salvage radiotherapy.

**Conclusions:**

Based on our findings, we propose a treatment strategy for biochemical recurrent disease after radical prostatectomy. Patients with Gleason score ≤ 7, positive surgical margin, and PSA velocity < 0.38 ng/mL/year are categorized the most favorable group, so that eradication by salvage radiation monotherapy could be expected. Other patients could be divided to two groups depending on surgical margin status and PSA velocity: 1) patients who might require combination of SRT and short-term androgen deprivation therapy and 2) patients who should be treated by androgen deprivation monotherapy.

## Introduction

Radical prostatectomy is selected as the initial therapy in 44% of American men with prostate cancer (PCa) [1]. Our data from Nara Uro-Oncological Research Group (NUORG) in Japan show an RP rate of approximately 30% (40% in stage cT1-2N0M0) with rates of radiation therapy, [2] but the proportion of radiation therapy is increasing [3, 4]. In case of stage cT1-2N0M0 PCa, the corresponding figure was 40%es. Although RP provides excellent cancer control, 15% to 40% of these men will experience recurrent disease within 5 years presenting as an increasing serum prostate-specific antigen (PSA) level without radiographic evidence of cancer [5–8]. Extraprostatic extension, seminal vesicle invasion and positive surgical margins are widely recognized risk factors of recurrence after RP. Three large randomized trials evaluating the role of post-RP adjuvant radiotherapy for PCa with these risk factors have been completed so far [9–11]. Adjuvant radiotherapy for patients with high-risk PCa provides significant improvement of biochemical recurrence (BCR)–free survival and clinical recurrence–free survival. These results have raised debate as to whether all patients with the risk factors should receive immediate adjuvant radiotherapy, or whether close surveillance and salvage radiotherapy (SRT) performed as soon as BCR is detected can provide a similar benefit and avoid overtreatment of men in whom disease does not progress. SRT is the only salvage therapy that can potentially achieve long-term freedom from BCR and clinical progression [12–15].

A critical issue in the management of these patients is to determine whether a rising serum PSA level after RP represents isolated recurrence of the surgical prostate bed or occult remote metastases that are undetectable by imaging. The former can potentially be eradicated by SRT. For the best chance of success, SRT of the surgical site must be administered when the recurrent tumor is localized and the tumor burden is low. Androgen-deprivation therapy (ADT) seems to provide only palliation to patients with recurrent prostate cancer.

To evaluate the benefit of SRT, we retrospectively reviewed a patient cohort of men who had undergone SRT monotherapy for the treatment of BCR after RP. We assessed their BCR-free survival to identify which subgroup had the greatest benefit from SRT, which subgroup needed combination therapy with SRT and ADT, and which subgroup received no benefit from SRT. To our knowledge, this is the first study in which three different groups were set based on the outcome of SRT to assess the clinicopathological background and detect predictive factors for the outcome of SRT.

## Materials and methods

### Patients

There were 94 consecutive patients who underwent SRT for the treatment of BCR after RP from January 2008 to December 2012 at Nara Medical University Hospital. Among them, 33 patients (35.1%) received ADT before the completion of SRT, including neoadjuvant therapy prior to RP and adjuvant therapy after RP and were excluded from analysis. Patient allocation was not randomized, but depended on the clinician’s decision. Thus for this study, we retrospectively reviewed the medical records of 61 patients who did not undergo ADT before the completion of SRT monotherapy. Medical records were reviewed for relevant clinicopathological information. Gleason score on prostate biopsy and radical prostatectomy specimen reported tumor grade. The 2002 TNM classification was used for staging [16]. Furthermore, patients were characterized into prognostic risk groups based on the NCCN classification system [17]. In all patients, digital rectal examination, pulmonary and abdominal computed tomography, and bone scans were performed before the initiation of SRT. No patients had any findings suggesting of distant metastases.

PSA doubling time (PSADT) and PSA velocity (PSAV) between the post-prostatectomy PSA nadir and the initiation of SRT was calculated using at least two PSA measurements with a 3-month interval and log calculations on the website of the Memorial Sloan Kettering Cancer Center (http://nomograms.mskcc.org/Prostate/PsaDoublingTime.aspx).

The protocol for the research project was approved by the Institutional Review Board for Clinical Studies (Medical Ethics Committee) and the study was conducted in compliance with the protocol and in accordance with the provisions of the Declaration of Helsinki (2010).

### Salvage radiotherapy (SRT)

SRT was defined as local radiotherapy to the prostatic bed alone following BCR after RP. All patients managed were seen at Nara Medical University Hospital and underwent simulation prior to treatment with three-dimensional conformal radiotherapy (3D-CRT) techniques with the treatment fields encompassing the prostatic and seminal vesicle bed plus periprostatic tissues. No attempt was made to comprehensively irradiate pelvic lymph nodes. Per our protocol, 70 Gy was delivered in daily fractions of 2.0 Gy. The fields were shaped to protect the small bowels, bladder, and posterior rectal wall.

### Post-SRT evaluation

**Figure 1:**
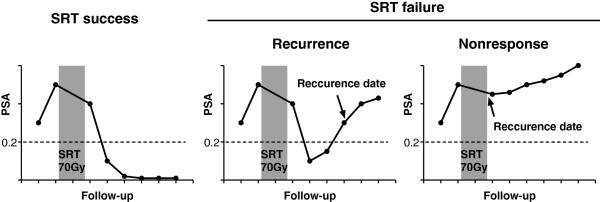
**Changes in serum PSA levels of patients who underwent salvage radiotherapy (SRT) for recurrent disease after radical prostatectomy.** Patients were evaluated by PSA measurement at regular intervals after completing SRT. Patients who showed a decline to PSA < 0.2 ng/mL after SRT and maintained PSA < 0.2 ng/mL during the follow-up were categorized as the SRT success group (left). Patients who showed a decline to PSA < 0.2 ng/mL after SRT and thereafter experienced a rise in PSA at two consecutive measurement points with the last PSA ≥ 0.2 ng/mL (middle) were categorized as the recurrence group. If the PSA continued to rise without a decline to PSA < 0.2 ng/mL after SRT, patients were categorized as the nonresponse group (right). The recurrence group and the nonresponse group were defined as the SRT failure group.

After SRT was completed, patients were evaluated by measuring PSA every 3 to 4 months for 5 years, and every 6 to 12 months thereafter. Median follow-up period was 29.6 months (12.0 - 70.0). The primary endpoint of this study was BCR after SRT. We divided the 61 patients into three groups according to the BCR status and the progress of treatment (Figure 1). The first group was defined as the SRT success group, and consisted of patients who showed a decline to PSA < 0.2 ng/mL after SRT and maintained PSA < 0.2 ng/mL during the follow-up (Figure 1, left). The second group was defined as the recurrence group, and consisted of patients who showed a decline to PSA < 0.2 ng/mL after SRT, and then experienced a rise in PSA at two consecutive measurement points to PSA ≥ 0.2 ng/mL at the last two measurement points (Figure 1, middle). The BCR date was defined as the first date with PSA ≥ 0.2 ng/mL in the recurrence group. The third group was defined as the nonresponse group. If the PSA continued to rise without a decline to PSA < 0.2 ng/mL after SRT, it was considered as nonresponse to SRT (Figure 1, right). The BCR date was the date of the completion of SRT in the nonresponse group. The recurrence group and the nonresponse group were categorized as the SRT failure group (Figure 1, middle and right). All endpoints were calculated from the date of the completion of SRT.

### Statistical analysis

Student’s t-test, Mann Whitney U-test, the chi-square test, and Fisher’s exact test were used to analyze the clinicopathological variables. The correlation between PSAV and PSADT was performed with Spearman’s correlation analysis. BCR-free survival curves were plotted according to the Kaplan Meier method, and the log-rank test was applied for statistical significance. We used multivariate Cox proportional hazards regression models of several clinicopathological variables to identify independent predictors of a poor response to SRT. The association was evaluated using the odds ratio (OR) and 95% confidence interval (CI) derived by standard logistic regression methods. For multivariate analysis, variables were selected on the condition that the P value was less than 0.1 in the univariate analysis. P values less than 0.05 were considered statistically significant. Statistical analysis was performed with SPSS Statistical Package version 20.0 (SPSS Inc., Chicago, IL).

## Results

**Figure 2:**
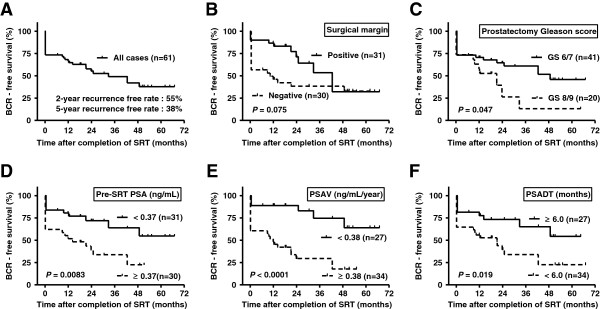
**Overall biochemical recurrence (BCR)-free survival.** Kaplan-Meier estimates of BCR in all patients **(A)**. Kaplan-Meier estimates of biochemical recurrence (BCR)-free survival by prostatectomy Gleason score **(B)**, surgical margin status **(C)**, pre-SRT PSA **(D)**, PSA velocity **(E)**, and PSA doubling time **(F)**. Survival curves are compared using the log rank test. The time to biochemical recurrence is given in months.

A total of 31 (50.8%) out of 61 patients showed BCR after SRT. The SRT failure group consisted of 16 patients in the recurrence group and 15 patients in the nonresponse group. The BCR free-survival at 2 and 5 years, respectively, was 55% and 38% (Figure 2A). No patients died of prostate cancer, but one patient died of lung cancer during the follow-up. Grade 3 and 4 adverse events after SRT were not observed. All patient characteristics are listed and the statistical comparison of the SRT success and SRT failure groups is shown in Table 1. There were no differences between the SRT success group and the SRT failure group according to age, initial PSA, risk classification, prostatectomy Gleason score (GS), pathological T stage, lymphatic invasion (ly), perineural invasion (pn), or surgical margin status using contingency table analysis (Table 1). However, univariate survival analysis demonstrated that BCR after SRT had a significant association with higher GS (Figure 2B, P = 0.047), while positive surgical margin approached significance (Figure 2C, P = 0.075). Among the PSA-related continuous values, pre-SRT PSA and PSAV were significantly higher and PSADT was significantly shorter in the SRT failure groups compared to the SRT success groups. The optimal cutoff of pre-SRT PSA, PSAV, and PSADT, respectively, was set as 0.37 ng/mL, 0.38 ng/mL/year, and 6.0 months, by testing all the data points yielding the highest p-value in each intergroup comparison (Figure 2D-F). All three values could be strong predictive parameters of BCR after SRT over time. Since Spearman’s correlation analysis showed that there was a high correlation between PSAV and PSADT (P < 0.0001; r = -0.74, 95% confidence interval -0.84 to -0.60) and PSAV was a better predictor than PSADT (Figure 2E and F), PSAV was selected for multivariate survival analysis. Multivariate Cox proportional hazard analysis revealed that BCR was significantly associated with GS ≥8 (HR 2.57, 95% CI 1.12-5.89), a negative surgical margin (HR 2.39, 95% CI 1.02-5.59), and PSAV ≥0.38 ng/mL/year (HR 4.44, 95% CI 1.58-12.5) (Table 2). In the most favorable subgroup of patients with GS ≤7, positive surgical margin, and PSAV < 0.38 ng/mL/year, only one of 10 cases (10%) experienced BCR after SRT, while in the least favorable group of patients GS >8, negative surgical margin and PSAV >0.38 ng/mL/year, all the four cases experienced BCR after SRT.

**Table 1:** **Characteristics of 61 patients undergoing SRT and comparison of SRT success group and SRT failure group**

Variables	Total (n = 61)	SRT success (n = 30)	SRT failure (n = 31 )	SRT Success ***vs***SRT failure ***P***value
**Age at SRT (median, range)**	**69 (57–78)**	**70 (58–77)**	**69 (57–78)**	**0.88 †**
**Initial PSA (mean, SD)**	**12.5 ± 14.5**	**14.3 ± 19.7**	**10.7 ± 6.4**	**0.96 †**
**Clinical T stage**				**0.47 ‡**
**T1c**	**24**	**13**	**11**	
**T2**	**29**	**12**	**17**	
**T3**	**8**	**5**	**3**	
**NCCN risk classification**				**0.086 ‡**
**Low**	**10**	**8**	**2**	
**Intermidiate**	**28**	**11**	**17**	
**High / Very high**	**23**	**11**	**12**	
**Prostatectomy Gleason score**				**0.17 ‡**
**6**	**6**	**5**	**1**	
**7**	**35**	**18**	**17**	
**8**	**8**	**2**	**6**	
**9**	**12**	**5**	**7**	
**Prostatectomy pathological T stage**				**0.84 ‡**
**T2a**	**10**	**6**	**4**	
**T2b**	**10**	**4**	**6**	
**T2c**	**14**	**7**	**7**	
**T3a / b**	**27**	**13**	**14**	
**Prostatectomy lymphatic invasion (ly)**				**0.23 ‡**
**Negative**	**46**	**25**	**21**	
**Positive**	**15**	**5**	**10**	
**Prostatectomy perineural invasion (pn)**				**0.79 ‡**
**Negative**	**23**	**12**	**11**	
**Positive**	**38**	**18**	**20**	
**Prostatectomy Seminal vesicle involvement**				**0.97 ‡**
**Negative**	**57**	**28**	**29**	
**Positive**	**4**	**2**	**2**	
**Surgical margins**				**0.075 ‡**
**Negative**	**30**	**11**	**19**	
**Positive**	**31**	**19**	**12**	
**Time to PSA nadir after prostatectomy (months)**	**2.4 (0.8 - 17.9)**	**2.3 (0.8 - 15.8)**	**2.5 (0.9 - 17.9)**	**0.22 †**
**PSA nadir after prostatectomy (ng/mL)**	**0.140 ± 0.238**	**0.116 ± 0.214**	**0.163 ± 0.262**	**0.35 †**
**Pre-SRT PSA (ng/mL)**	**0.56 ± 0.49**	**0.37 ± 0.25**	**0.75 ± 0.59**	**0.021 †**
**PSAV after PSA recurrence (ng/mL/year)**	**0.19 ± 1.40**	**0.38 ± 0.40**	**1.43 ± 1.79**	**< 0.0001 †**
**PSADT after PSA recurrence (months)**	**7.44 ± 7.27**	**9.89 ± 9.02**	**5.07 ± 3.94**	**0.0049 †**
**Follow up period after SRT (months)**	**29.6 (12.0 - 70.0)**	**24.6 (12.0 - 70.0)**	**31.2 (12.0 - 64.6)**	**0.83 †**

SRT = salvage radiotherapy; PSA = prostate-specific antigen; SD = standard deviation; NCCN = The National Comprehensive Cancer Network; PSAV = PSA velocity; PSADT = PSA doubling time; † Man-Whitney U test; ‡ Chi-square test or Fisher’s exact test.

**Table 2:** **Cox proportional models for SRT failure risks**

Variables	HR	95% CI	***P***value
**Prostatectomy Gleason score**			
**6 / 7**	**1**		
**8 / 9**	**2.57**	**1.12 - 5.89**	**0.026**
**Surgical margin**			
**Positive**	**1**		
**Negative**	**2.39**	**1.02 - 5.59**	**0.045**
**Pre-SRT PSA (ng/mL)**			
**< 0.37**	**1**		
**≥ 0.37**	**1.18**	**0.48 - 2.89**	**0.71**
**PSAV (ng/mL/year)**			
**< 0.38**	**1**		
**≥ 0.38**	**4.44**	**1.58 - 12.5**	**0.005**

HR = Hazard ratio; CI = confidence interval; SRT = salvage radiotherapy; PSAV = PSA velocity.

**Figure 3:**
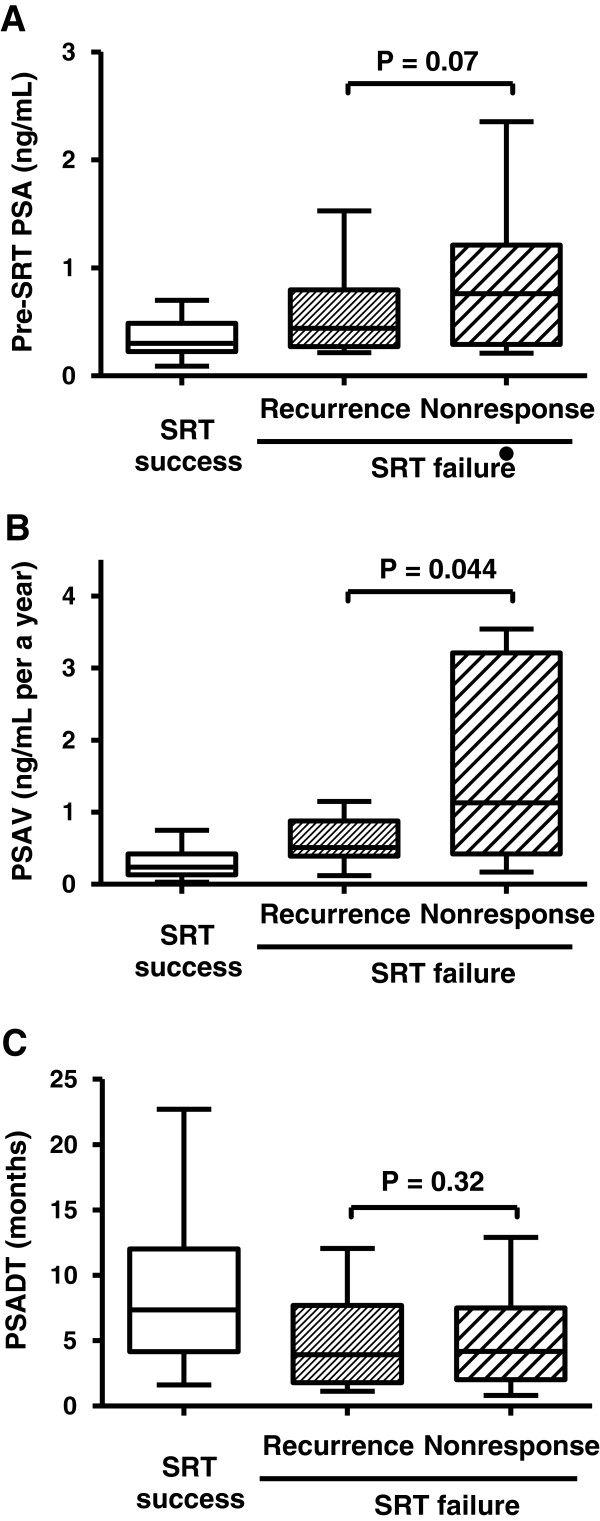
**Comparison of PSA-related values between the SRT success group, recurrence group and nonresponse group.** Pre-SRT PSA **(A)**, PSAV **(B)**, and PSADT **(C)** were depicted by Tukey box plots. Horizontal lines within boxes indicate median levels and are depicted by horizontal lines within boxes. Significance (P < 0.05) was assessed by the Mann–Whitney U test.

Next, we set out to determine which clinicopathological parameters which could distinguish between the recurrence group and the nonresponse group within the SRT failure group. Three PSA-related values associated with BCR after SRT were compared between the recurrence group and the nonresponse group (Figure 3). While the PSADT did not show any differences, there was a significant difference in PSAV (P = 0.044) and a marginal difference in pre-SRT PSA (P = 0.07). In a total of 31 cases consisting of 16 cases in the recurrence group and 15 cases in the nonresponse group, we determined optimal cut-off values of pre-SRT PSA and PSAV using a dichotomous test. Pre-SRT PSA ≥0.5 ng/mL and PSAV ≥0.8 ng/mL/year were significant parameters distinguishing the nonresponse group from the recurrence group (Table 3). Univariate analysis revealed that only negative surgical margin (odds ratio = 5.14; P = 0.038) was associated with nonresponse to SRT. Multivariate logistic regression analysis also identified negative surgical margin (odds ratio = 12.71; P = 0.039) and PSAV ≥0.8 ng/mL/year (odds ratio = 12.14; P = 0.039) as independent predictors of a poor response to SRT.

**Table 3:** **Univariate and multivariate analysis of factors in the SRT failure group that distinguish the nonresponse group from the recurrence group**

Variables	Univariate analysis			Multivariate analysis †		
	OR	95% CI	***P***value	OR	95% CI	***P***value
**Prostatectomy Gleason score**						
**6 / 7**	**1**					
**8 / 9**	**0.5**	**0.12 - 2.14**	**0.35**			
**Prostatectomy lymphatic invasion (ly)**						
**Negative**	**1**			**1**		
**Positive**	**3.79**	**0.75 - 19.1**	**0.097**	**2.95**	**0.30 - 29.4**	**0.36**
**Prostatectomy perineural invasion (pn)**						
**Negative**	**1**			**1**		
**Positive**	**4.00**	**0.81 - 19.8**	**0.081**	**3.56**	**0.47 - 26.8**	**0.22**
**Surgical margin**						
**Positive**	**1**			**1**		
**Negative**	**5.14**	**1.03 - 25.6**	**0.038**	**12.71**	**1.14 - 142.1**	**0.039**
**Pre-SRT PSA (ng/mL)**						
**< 0.5**	**1**					
**≥ 0.5**	**3.33**	**0.76 - 14.6**	**0.11**			
**PSAV (ng/mL/year)**						
**< 0.8**	**1**			**1**		
**≥ 0.8**	**4.13**	**0.88 - 19.3**	**0.065**	**12.14**	**1.13 - 129.9**	**0.039**

OR = Odds ratio; CI = confidence interval; SRT = salvage radiotherapy; PSAV = PSA velocity; † Logistic regression analysis.

## Discussion

Approximately one third of men undergoing RP for curative intent of their PCa will experience BCR within 5 years following RP [5–9]. If salvage therapy is withheld, two-thirds of recurrent cases will develop bone metastases within 10 years of BCR [18]. Salvage therapies with the intent to cure after BCR PCa consist of SRT and SRT with concomitant ADT. ADT along with SRT or as monotherapy should only be selected in cases that cannot be cured with SRT monotherapy, because ADT can have deleterious effects on the quality of life, increased risks for serious health concerns, and psychological distress [19, 20]. However, there are uncertainties about the optimal timing of SRT after RP (adjuvant setting or salvage setting), especially in ethnic groups that were not represented well in the three randomized trials on post-operative radiation after RP [9–11].

In this study, we retrospectively reviewed a patient cohort of men who had undergone SRT monotherapy to identify prognostic factors for BCR after SRT. Our findings demonstrated that GS 8 or more, negative surgical margin, and PSAV ≥0.38 ng/mL/year were independent predictive factors for poor SRT sensitivity (Table 3). Only one of 10 cases (10%) with three favorable factors (GS ≤7, positive surgical margin, and PSAV <0.38 ng/mL/year) experienced BCR after SRT, while all of four cases with the three adverse factors (GS ≤ 8, negative surgical margin and PSAV ≥0.38 experienced BCR after SRT. A consensus report from the Genito-Urinary Radiation Oncologists of Canada (GUROC) show that a number of factors could predict disease progression: high GS, short PSADT, high pre-SRT PSA, negative surgical margin, and seminal vesicle involvement at the time of RP [15].

In most previously published studies, patients who were treated with ADT before the completion of SRT, such as a neoadjuvant agents or adjuvant agents in association with RP, were enrolled. Statistics can be biased by the inclusion of patients who had undergone ADT before the completion of SRT if disease progression is defined as a rise in PSA. In our study, we excluded 33 patients who were treated with ADT before the completion of SRT, yielding 61 patients who were treated with SRT monotherapy for recurrent disease after RP. The exclusion enabled a unique analysis and identification of clincopathological factors predicting SRT-success, recurrence, and nonresponse groups.

**Figure 4:**
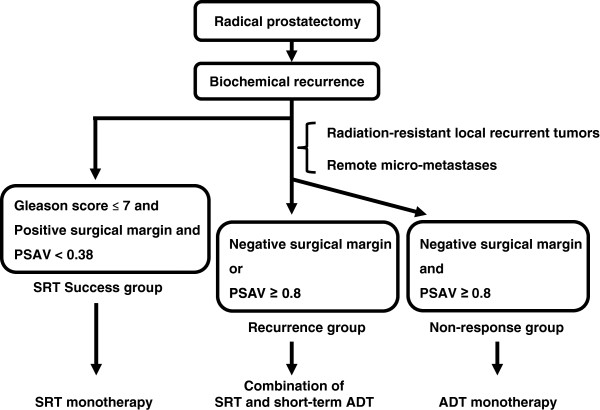
**Proposed treatment strategy for recurrent disease after radical prostatectomy.** The OR that patients with three favorable factors will experience BCR after SRT monotherapy is 0.084 (95% CI 0.01-0.72). Patients showing biochemical recurrence after a decline to PSA < 0.2 ng/mL and those showing nonresponse to SRT can be distinguished by the status of the surgical margin and PSAV. The former may benefit from the combination of SRT and short course of ADT, while the latter may not receive benefit from SRT.

SRT monotherapy should be selected for patients with favorable factors while ADT alone could be selected for patients in whom eradication of the recurrent disease after RP is unlikely. Figure 4 shows a proposed treatment strategy based on our findings. In patients harboring three favorable factors including low GS, positive surgical margin, and low PSAV, eradication by SRT monotherapy can be expected. In contrast, in patients who do not meet the conditions of favorable factors, it can be suspected that radiation-resistant local recurrent tumor, remote micro-metastases, or both may develop. It means that in patients without the three favorable factors, SRT monotherapy will likely prove inadequate to eradicate recurrent disease. Thus these patients can be divided according to the treatment progress into two groups: those showing biochemical recurrence after a decline to PSA < 0.2 ng/mL, and those presenting with nonresponse to SRT. The former (positive surgical margins or PSAV < 0.8) predominantly includes patients who are unable to be salvaged by SRT monotherapy and who require combination with short-term ADT. The latter (negative surgical margins and PSAV ≥ 0.8) predominantly includes patients who do not benefit from SRT and should be treated by ADT monotherapy.

There are several limitations of the current study including it being a retrospective study design with a small sample size from a single-center and there being selection bias. Furthermore, the study lacks overall survival and cause-specific survival analysis due to the short follow-up period. Validation of these results in future prospective multi-center studies will be needed.

## Consent

Written informed consent was obtained from the patient for the publication of this report and any accompanying images.

## Abbreviations

RP: Radical prostatectomy
SRT: Salvage radiotherapy
PSA: Prostate-specific antigen
PCa: Prostate cancer
BCR: Biochemical recurrence
ADT: Androgen-deprivation therapy
PSADT: PSA doubling time
PSAV: PSA velocity
GS: Gleason score.
